# Significant effects: A personal perspective on how health psychologists can influence policy and practice

**DOI:** 10.1111/bjhp.12210

**Published:** 2017-04-01

**Authors:** Robert West

**Affiliations:** ^1^ Cancer Research UK Health Behaviour Research CentreUniversity College LondonUK

## Background

Health psychology should have important things to say to policymakers and practitioners in health care. It addresses questions of vital importance to the health of the population. What policies will give the best results when trying to encourage and support people to lead healthier lives? How can we best help people manage chronic diseases? How can we help people cope emotionally with illness and death? What can be done to improve adherence of health professionals to good practice guidelines? How can we reduce the rates of medical accidents and misdiagnoses?

## Having something to say

Health psychology has more useful answers to these questions than can be arrived at by ‘common sense’, and every day new findings are being published which provide greater insights. It is wasteful and counterproductive for policies to be enacted that fail to take account of these insights. Practice can be slow to take account of research findings in most or all areas of human endeavour, and sometimes it never does. This article draws on my own experience to consider how health psychologists can improve the impact our findings have on practice. It is a personal perspective and other people's experience may differ, but hopefully it will provide some useful pointers.

## Who will listen?

My experience is that when the conditions are right, policymakers and practitioners are surprisingly keen to pay attention to health psychology and indeed, their appetite for findings and advice can outstrip our ability to deliver these. The two defining conditions for this appear to be as follows: (1) when they feel a need for academic input to help them reach their professional objectives and (2) when they have academics they feel they can trust and want to work with. Timeliness is often important here – political and policy agendas can change very quickly, and it is important to be ready to assist when the need arises. It seems that policymakers and practitioners are disinclined to listen to expert academic input when they are under political pressure to pursue a line regardless of the evidence, they need to act quickly, or they already have trusted sources (even though these may not have the required expertise).

Table 1 lists my personal experience of the main types of organization that engage with health psychologists, with examples and the kinds of work that can be involved. It can range from being available on the end of the telephone to answering queries or pointing to evidence in the literature, to setting up or being part of organizations as a trustee. The types of organization range from very local to international and can embrace for‐profit companies, charities, and public sector bodies.

**Table 1:** Types of engagement with policymakers and practitioners

Type of organization	Examples	Types of activity
International agencies and policymakers	World Health Organization World Bank International Union Against Cancer European Monitoring Agency for Drugs and Drug Addiction European Respiratory Society Bloomberg Foundation Gates Foundation European Commission Foreign Government Ministers	Guideline development Cost‐effectiveness estimation Training Preparing evidence reviews Personal discussion Preparing legal submissions
National government and governmental agencies and policymakers	Government Ministers Public Health England Department of Health National Institute of Health and Care Excellence All Party Parliamentary Groups Parliamentary Select Committees	Guideline development Cost‐effectiveness estimation Training Preparing evidence reviews Personal discussion Preparing legal submissions Attending hearings
Local government organizations	Local Authorities Local Government Association Directors of Public Health Local political parties Local councillors	Writing reports Conducting research Undertaking evaluations Speaking at meetings
Charities	Action on Smoking and Health QUIT The Tommy's Campaign	Providing advice Writing reports Taking on role of trustee Helping with funding applications
Public sector service providers	National Health Service Trusts Individual clinics and clinicians	Providing advice Developing interventions Training
Private service providers	Quit 51 Solutions for Health National Centre for Smoking Cessation and Training	Advisory board membership Collaboration Treatment manual development Training
Product developers and manufacturers	Pharmaceutical companies Start‐up companies	Advisory board membership Report writing Providing training Acting as an expert witness Helping with dissemination of findings Training
Media	Broadcast media Newsprint Social media	Attending press conferences Writing articles Doing interviews Advising on and contributing to documentaries

In some cases, such as sitting on committees, a level of seniority seems to be a prerequisite for getting involved, but in most instances, this is not the case. Credibility as a researcher stems from having something useful to say and saying it clearly.

## How to get involved

Opportunities to get involved arise frequently. There are calls from bodies such as NICE to sit on guideline development panels and invitations from organizations as a result of talks one has given or papers one has published. Often, whatever one's level of seniority, opportunities arise through personal contacts or recommendations. Very often involvement in one type of engagement leads to opportunities to get involved in others. If one is entrepreneurial, one can relatively easily set up organizations such as community interest companies to deliver services or provide health care products arising from one's research.

When it comes to involvement with the media, academic journals and universities are increasingly keen to issue press releases to publicize findings. Often journalists are looking for academics to comment on findings. A particularly important organization in the United Kingdom for disseminating and commenting on research findings is the Science Media Centre (www.sciencemediacentre.org). This is a charity whose purpose is to improve the reporting of science, and it does it very well. If one has a particularly important or interesting finding, it is worth contacting them to see whether they would be willing to host a press conference on it.

## Why get involved

While, at a personal level, it can be satisfying to know that one's findings are considered useful, in my view, the only legitimate reason for getting involved is to make a positive difference to people's lives. Policy and practice should be based on a dispassionate analysis of the best available evidence. My impression is that much of it is not. This can be because of ignorance or bias on the part of policymakers. Unfortunately, it can also be because of ignorance, incompetence, or bias on the part of academics advising them. An important reason for getting involved in policy and practice debates is to counteract this misinformation.

## Not getting carried away

When we have the ear of policymakers and practitioners, it is crucial that health psychologists respond appropriately – being receptive to invitations and interpreting findings accurately and communicating them dispassionately in a way that is understood and usable. It is also important for academics to remain detached from the specific goals of those with whom we are working and keep in sight the broader scientific and health agenda.

The UK Department of Health's Chief Scientific Officer has set out a number of principles for academics to follow to maximize the benefit of their work for policymakers, and one of these is to refrain from making policy recommendations when writing (Whitty, 2015). Policy development requires a level and type of analysis that goes far beyond what the author of an academic paper typically has the competence to undertake. Our role as academics is to try to ensure that whatever policy decisions are made, they have full cognizance of the evidence and the implications of this for the effects of the options being considered. This is not to say that as concerned citizens, we may not express preferences or try to persuade policymakers or practitioners to go down particular paths, but mixing this up with our role as advisors undermines our credibility as scientists and risks straying into areas outside of our competence.

## Developing relevant skills

Communicating scientific findings to policymakers and practitioners requires a particular set of skills. Like any skill, these need to be acquired and honed. I have found that when it comes to applying those skills, it helps to focus on the following: (1) stick to what is clearly relevant and (2) apply the motto: keep things simple, don't make them simple.

With regard to the first of these, it is all too easy to go down the road of arcane discussions about points of methodology or theory, or to offer fine distinctions that make little difference in practice.

A corollary of this is that we must not be afraid to admit ignorance. Many questions cannot be answered by the available evidence, and our own personal areas of expertise are limited. In the field of public health, in particular, I have found that some high profile public health experts offer strong opinions on topics about which they lack expertise. This can be particularly harmful, both by misleading policymakers and the public and in undermining the credibility of public health as a discipline.

It is also usually unhelpful to answer policy questions with a simple ‘it depends’. We can usually do better than that, providing an indication of what might be expected in what circumstances, duly qualified in terms of the basis on which the judgement is being made.

For the second goal, it is crucial to strive to convey findings as simply as possible but not to oversimplify. In public health, there is an unfortunate tendency for some academics to present findings in simplistic ways that are highly misleading. It is tempting, when engaging with policymakers and practitioners, to disregard the caveats we put into scientific papers about not inferring causal associations from correlations.

## Staying honest

When one establishes an ongoing relationship with an organization or set of individuals in an organization, it is easy to fall into the trap of taking on their agenda. For example, when sitting on an advisory board of a pharmaceutical company, it is easy to begin to see things in terms of the commercial success of their products. This can be because we come to believe in those products, as a natural extension of one's role, or because we come to like the people in the company that we are working with. It is crucial to guard against this and always keep in mind one's duty as a publicly funded scientist.

There may be active pressure to engage in public relations and marketing for organizations we are working with. In my view, it is important to avoid this unless it is for a public sector organization where there is no financial motive. Even being on the panel at a press conference launching a new product can be problematic, with the academic becoming identified with the product.

Whether or not we think we have been influenced by a relationship with an organization, financial or otherwise, the fact is that we probably are. It is therefore essential to ensure that in all our dealings and writing, we are open about our potential competing interests. Journals and some conferences require us to declare financial conflicts of interest, but in my experience, bias is even harder to avoid from ones relating to values and relationships with organizations or individuals. So in my view, even non‐financial relationships with organizations that have a vested interest in research outcomes should be declared.

## Getting shot at

Being willing to get involved in policy and practice almost inevitably means coming under fire from people who disagree with one's viewpoint or analysis, either because of their own vested interests, personal prejudice, or simply that they read the evidence differently. Sometimes, the attacks can be personal and hostile. One should always be willing to consider the possibility that one has got it wrong and to correct mistakes or revise one's opinions. However, one also needs to have the mental strength to defend a position in the face of opposition, sometimes from one's colleagues.

In all of this, I find it invaluable to use my network of friends and colleagues as a sounding board. Did I get it right? What have I not considered? Should I respond and if so how? It is rare that one is completely isolated in a particular area and one's contribution is often as part of a collective. In those cases, it is important for the collective to act together in dealing with attacks.

## Is it all worth it?

I estimate that I spend about 10–15% of my time on matters related to policy and practice. That is time that I could spend either doing purely academic work or playing guitar. The work can provide intellectual and personal satisfaction, but the main reason for doing it is the belief that it can make a positive difference – not only in terms of translation of research into practice but also informing the research agenda and providing valuable scientific insights.

## Conflict of interest

The author undertakes research and consultancy for companies that develop and manufacture smoking cessation medications (Pfizer, J&J and GSK). He is an unpaid advisor to the UK's National Centre for Smoking Cessation and Training. His salary is funded by Cancer Research UK. He is a member of the Labour Party.
